# The Proposed Congress of American Physicians and Surgeons and How It Will Affect the American Medical Association

**Published:** 1886-07

**Authors:** 


### Correspondence.

### THE PROPOSED CONGRESS OF AMERICAN PHYSICIANS AND SURGEONS AND HOW IT WILL AFFECT THE AMERICAN MEDICAL ASSOCIATION.

*Editors Atlanta Medical and Surgical Journal:*

The outlook of the American Medical Association calls for consideration at present in connection with the proposed Congress of American Physicians and Surgeons.

It does not require any great acumen to perceive that the disclaimer of any intention of "offering an obstacle or opposition to any other organization" on the part of the specialists, who are moving in this co-operative undertaking, is a blind to lull the suspicions of the orthodox members of the time-honored association of the medical profession in this country as to any foul play toward them and their work. The plea of convenience is urged for uniting the following named associations into a congress: The American Surgical Association, the American Ophthalmological Association, the American Otological Association, the American Neurological Association, the American Laryngological Association, the American Gynæcological Society, the American Dermatological Association, the American Climatological Association and the American Clinical and Pathological Association. The plan seems to ignore the Dental and Pharmaceutical Associations, which have been formed in different sections of our medical commonwealth; but of course these can be incorporated upon application subsequently, if not considered too commonplace for intercourse with "the members of the said special societies," who, it has been claimed, "were the representatives of the profession in America."

These specialists bearing presents are evidently expecting to repeat the ruse of introducing a Trojan horse with masked enemies to the great prejudice of medical progress in this country; and those who are not prepared as yet to recognize the complete sway of specialism in the profession should beware how they fall into this trap which has been laid for them by the special pleading of "the opening sessions of each annual meeting of the congress, to be devoted tc such general business as might pertain to the interests of the Association as a whole."

It is not expected that anything connected with the interests of general practitioners throughout the United States shall enter into the programme of such a congress, and the large and respectable class of the profession, who are inclined to these lines of service to a greater or less extent, may be misled into becoming identified with this movement, to the detriment of the American Medical Association, to which they owe their allegiance, as affording the true basis of scientific medicine. A remark of the *Weekly Medical Review*, of St. Louis, in an entirely different connection is applicable to this case. There can be no doubt that the proper treatment of all irregular offshoots of the mother profession is the dignified ignoring of them, and it is to be hoped that none but those who desire to be classed as specialists shall fall into line under this distinctive flag. If only such elements compose this new congress, there will be no cause to complain by the regular members of the medical profession, who should congratulate themselves on their separation in a distinct organization, and "membership in the congress to be acquired only by virtue of fellowship in one or another of the special organizations."

Dr. R. A. Kinloch very properly expressed what should be the sentiment of all the members of the American Medical Association when he deprecated the tendency to specialties in that body at its recent meeting in St. Louis ; and while different departments of investigation are undertaken, there are no good and sufficient reasons why distinct sections in special divisions should be kept up in that organization intended for the general good of the whole medical profession. It is calculated to embarrass the ample and free discussion of topics of general interest that they should be referred to those separate coteries; and it was painfully evident in the morning sessions of the entire Association that matters were hurried up so as not to be satisfactory to those who went there for instruction and mutual profit. In a deliberative assembly, such as this Association, there should be no indecent haste and flurry manifested in the proceedings, and this desirable end would be attained by providing more time for the regular addresses in different departments, with an opportunity for discussions upon each by two or three appointed previously, and by volunteers afterwards, in speeches of ten minutes. A set address, prepared with care, is certainly entitled to due consideration and discussion by the members present, and practical points of importance are likely to be brought out in the interchange of views on the occasion of the meeting.

It would further seem very desirable that the afternoons should be devoted to voluntary papers in general session, thus affording an opportunity for capable men in the profession to come forward with useful contributions who would otherwise never be heard from, under the pre-assignment which now holds sway in the sections, as well as in the open meeting.

It is the grandest mistake of the age to infer that those who are brought to the front by appointment are the best qualified to furnish useful papers at the meetings of the American Medical Association, and there should at least be a fair showing for those who are not included in such assignments. The representative men of to-day may find that others are fitted to take their places to-morrow, and we should not block up the way to advancement on the part of meritorious candidates for the prize of championship in medicine.

These modifications in the programme of business are founded upon the supposition that there is much latent talent in the profession, not included in either of the various specialties, and that it would be developed by the elimination of specialism as proposed in this new congress.

